# Improving maternal and fetal pregnancy outcomes: in memoriam, Doctor Frederick Morfaw (1980-2020)

**DOI:** 10.1186/s40814-020-00663-5

**Published:** 2020-09-08

**Authors:** Lawrence Mbuagbaw, Lehana Thabane

**Affiliations:** 1 Department of Health Research Methods, Evidence and Impact, McMaster University, Hamilton, Ontario Canada; 2 Biostatistics Unit, Father Sean O’Sullivan Research Centre, St Joseph’s Healthcare-Hamilton, 50 Charlton Avenue East, 3rd Floor Martha Wing, Room H321, Hamilton, Ontario L8N 4A6 Canada; 3 Centre for the Development of Best Practices in Health, Yaoundé, Cameroon; 4 Departments of Pediatrics and Anesthesia, McMaster University, Hamilton, Ontario Canada; 5 Centre for Evaluation of Medicine, St Joseph’s Healthcare-Hamilton, Hamilton, Ontario Canada; 6 Population Health Research Institute, Hamilton Health Sciences, Hamilton, Ontario Canada

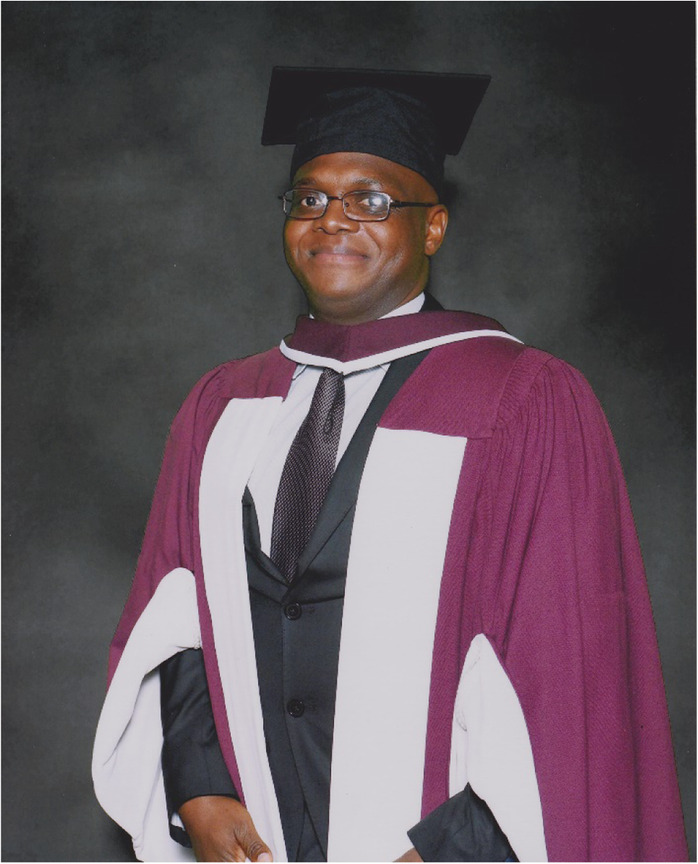



We are disheartened by the sudden passing of Dr. Frederick Morfaw. He was an avid advocate of mother and child health research and made important contributions to male partner participation in vertical transmission of HIV programs. Before his untimely death on Monday, June 15, 2020, he had just completed a research fellowship in perinatal clinical epidemiology with McMaster’s Department of Obstetrics and Gynecology. Born in Limbe, South West Region, Cameroon, on January 5, 1980, he pursued a life of academic excellence. He completed his secondary and high school at Sacred Heart College, Bamenda, and then trained as a Medical Doctor in the Faculty of Medicine and Biomedical Sciences, University of Yaoundé 1 from 1998 to 2005 graduating with a Doctor of Medicine (MD) degree. He then completed a Master of Science in Public Health Research at the University of Edinburgh in 2008. Following his return from the UK, he completed his specialization in Obstetrics and Gynecology in 2013 with honors at the University of Yaoundé I. He worked as a consultant gynecologist/obstetrician for 5 years at the Bamenda Regional Hospital. During this specialization, he completed an international postdoctoral fellowship with the Canadian HIV Trials Network in 2012. His accolades include the *Mandela Washington African Leadership Initiative award* (*2015*); *the Drug Safety and Effectiveness Cross-Disciplinary Training Program Award* (*2018*); *the Ontario Drug Policy Research Network Student Training Program Award* (*2019*); *and the* David L. Sackett Scholarship (2019)

## Contributions to science and to mother-and-child health

Frederick was a reviewer and associate editor for *Pilot and Feasibility Studies* since 2018 and personally oversaw the review and publication of several manuscripts, providing insight into how best to design, conduct, and report pilot studies in mother and child health. He completed important research on male partner involvement in prevention programs of mother to child transmission of HIV, highlighting the importance of having partners support their spouses morally and financially, in order to enhance the uptake of prevention programs [1]. His work led to the development of a scale that can be used to measure male partner participation. He also conducted research highlighting the benefits of misoprostol in preventing post partum hemorrhage (PPH) [2], which is responsible for one maternal death in low-income countries every 4 min [3].

Frederick was dedicated to mentoring junior colleagues. Numerous colleagues in Cameroon and Canada can attest to his highly supportive and collaborative approach to research. He contributed to building capacity in HIV research in Africa through workshops and also helped outline guidance for early career health researchers [4, 5]. He was part of a network of early career researchers supported by the Canadian HIV Trials Network that played an important role in building HIV research capacity on the African continent [6].

Frederick is survived by his wife, Laura, two daughters, Vanessa and Ricka, and one son, Prince Henry. He will be remembered for his cheerful hearty laughter and jovial disposition. In addition to his academic achievements, Frederick excelled in gymnastics as a child, played soccer, ping-pong, and took pleasure in traveling. We honor his life and legacy by continuing his research. Rest in peace, Frederick, our brother, friend, colleague, and mentor.

## Data Availability

Not applicable
